# Regulation of *MIR* Genes in Response to Abiotic Stress in *Hevea brasiliensis*

**DOI:** 10.3390/ijms141019587

**Published:** 2013-09-27

**Authors:** Virginie Gébelin, Julie Leclercq, Songnian Hu, Chaorong Tang, Pascal Montoro

**Affiliations:** 1 Centre de Coopération Internationale en Recherche Agronomique Pour le Développement, Unité Mixte de Recherche Amélioration Génétique et Adaptation des Plantes Méditerranéennes et Tropicales, Avenue Agropolis, Montpellier F-34398, France; E-Mails: gebelin.v@gmail.com (V.G.); julie.leclercq@cirad.fr (J.L.); 2 Beijing Institute of Genomics, Chinese Academy of Sciences, Beijing 100029, China; E-Mail: husn@big.ac.cn; 3 Rubber Research Institute, Chinese Academy of Tropical Agricultural Sciences, Danzhou 531737, China; E-Mail: chaorongtang@126.com

**Keywords:** gene expression, miRNA, *MIR* gene, abiotic stress, rubber tree, tapping panel dryness

## Abstract

Increasing demand for natural rubber (NR) calls for an increase in latex yield and also an extension of rubber plantations in marginal zones. Both harvesting and abiotic stresses lead to tapping panel dryness through the production of reactive oxygen species. Many microRNAs regulated during abiotic stress modulate growth and development. The objective of this paper was to study the regulation of microRNAs in response to different types of abiotic stress and hormone treatments in *Hevea*. Regulation of *MIR* genes differs depending on the tissue and abiotic stress applied. A negative co-regulation between *HbMIR398b* with its *chloroplastic HbCuZnSOD* target messenger is observed in response to salinity. The involvement of *MIR* gene regulation during latex harvesting and tapping panel dryness (TPD) occurrence is further discussed.

## 1. Introduction

*Hevea brasiliensis* is the only source of natural rubber (NR), which is produced in latex cells in the phloem. Latex harvesting consists in tapping the soft bark of rubber trees. Ethephon is applied to stimulate production. Increasing demand for NR calls for an increase in latex yield, but also an extension of crops to marginal zones. Such marginal zones are subject to stronger and more detrimental abiotic constraints for latex production (positive cold, frost-prone, drought, *etc.*). Exposure to abiotic stress in addition to latex harvesting stress affects latex production and tree productivity [1]. Laticifer cells are the site of numerous types of stress that lead to oxidative stress. The plant’s first response to wounding is to produce jasmonate [2]. The wound and associated jasmonate production also cause oxidative stress in the plant and activate antioxidant systems to overcome it [3]. Oxidative stress, whether produced in response to tapping and/or environmental constraints, may lie behind problems linked to latex flow [4]. It is reflected in an imbalance between ROS (reactive oxygen species) and the detoxification systems existing in laticifers. ROS then attack the membranes of cell organelles, causing the release of coagulant factors existing in the lutoids, which gives rise to *in situ* coagulation of rubber particles. This complex physiological disorder is called TPD (Tapping Panel Dryness). This phenomenon remains reversible up to a certain limit. It amounts to laticifer fatigue, called dry cut. In extreme cases, halted flow is followed by the degenerescence and death of the laticifers and, consequently, severe cortical necrosis (Brown Bast) [5]. Annual losses due to TPD are estimated at 10% to 40% [1].

MicroRNAs (miRNAs) form a small class of non-coding RNAs of 21 to 24 nucleotides involved in numerous physiological processes in plants including response to biotic and abiotic stresses through post-transcriptional regulation. miRNA biogenesis requires several stages and several enzymes from *MIR* gene transcription to the generation of mature miRNA [6–10]. The mature miRNA enters the complex called RISC (RNA-induced silencing complex) thereby enabling regulation of the target gene. In the RISC complex, the mature miRNA associates with proteins of the Argonaute family (AGO) to direct the regulation of the target gene [11,12]. In the RISC complex, the mature miRNA sequence links up with the sequence of the messenger RNA through perfect or imperfect sequence homology. This action causes a silencing phenomenon in plants, called PTGS (post-transcriptional gene silencing) [13]. In plants, most target messenger RNAs contain a single site complementary to the miRNA, corresponding to the cleavage site [9], suggesting that plant microRNAs use RNA cleavage rather than preventing translation. Long microRNAs are taken in charge by Dicer-like 3 (DCL3), associate with AGO4 and then guide methylation of the DNA of target genes in rice and mosses [14,15].

Many microRNAs regulated by abiotic stress have been identified in the model species (for review, see [16]). The first involvements of microRNAs in response to stress were described by Jones Rhoades and Bartel who, in *Arabidopsis thaliana*, predicted genes targeted by microRNAs such as superoxide dismutase, the laccases and ATP sulfurylases (APS) [17]. MicroRNA expression was then studied in different contexts of abiotic stress in numerous species (for review, see [16]). Most microRNAs conserved between species target transcription factors, such as miR/target gene pairs *miR156/SBP*, *miR159/319/MYB-TCP*, *miR160/ARF*, *miR166/HDZIPIII*, *miR169/NFY* subunit.

MicroRNAs are also involved in the regulation of antioxidant activities and particularly in the regulation of reactive oxygen species-scavenging enzymes. The production of ROS is linked to abiotic and biotic stress status in plants [18–20] and the excess ROS can lead to several types of cell damage, such as peroxidation of membrane lipid compounds, degradation of polysaccharides, denaturing of enzymes and lesions in DNA [21]. The miR398 targets the *CSD1* and *CSD2* genes encoding cytosolic and chloroplastic CuZnSOD in *Arabidopsis thaliana* [22]. The level of expression of these microRNAs and of their target is regulated in response to stress, involving a modulation of growth and development during stress. For example, miR398 is under-expressed in response to stress which leads to an accumulation of CuZnSOD enabling detoxification of an excess of reactive oxygen species in *Arabidopsis thaliana* [22].

To date, 28 sequences of *MIR* are accessible in miRBase [23–25] for the rubber tree. They represent 10 conserved families and 14 newly identified families potentially *Hevea*-specific miR [26,27], added to which four additional precursors have been identified recently [28]. Our previous studies on mature trees demonstrated the functionality of miRNA biogenesis in the latex cells. Moreover, we highlighted the reduction of small RNA size from 24 to 21 nucleotides in TPD-affected trees, which could not be explained by a general RNA degradation [28]. From this previous work, we hypothesized that the change in size could also be effective for mature miRNAs and consequently involved to independent miRNA pathway, one leading to targeted transcript cleavage and the other to methylation of targeted genes (for review [29]). Among all pre-miRNAs tested, only the relative *Hbpre-MIR159b* abundance was up-regulated between TPD-affected and healthy trees. Targets of miR159 were predicted and might be involved in various activities such as rubber biosynthesis, antioxidant activity and transcription regulation activity [26,28,30].

The objective of this paper was to study the regulation of microRNAs in response to different types of abiotic stress (cold, saline stress, wounding) or hormone treatments (ethylene and methyl jasmonate). Given some mature miRNAs could be produced by several *MIR* genes, this study focused on the expression analysis pattern using real-time RT-PCR with specific primers for each *MIR* genes. The relative accumulation of 19 premiRs was analyzed by real-time RT-PCR on various tissues from juvenile plant material in order to facilitate the application of treatments. Negative co-regulation of *Hbpre-miR398b* and its target *chloroplastic CuZnSOD* is revealed in response to saline stress.

## 2. Results and Discussion

### 2.1. Relative Accumulation of premiR in Response to Abiotic Stress in *in Vitro* Plantlets

Relative accumulation of premiR of 20 selected *MIR* genes was analysed in different tissues (leaf, bark and root; Table 1). In control plants, the conserved *MIR* genes were generally very weakly expressed such as *HbMIR156*, *HbMIR319* and *HbMIR398a* and *HbMIR398b* (from 6.78 × 10^−4^ in roots for *HbMIR398a* to 9.22 × 10^−2^ for *HbMIR319* in bark). By contrast, the *HbMIR408a* and *HbMIR408b* genes were strongly expressed (from 8.47 × 10^−1^ to 3.54). Among the *MIR* genes newly identified in *Hevea brasiliensis*, *HbMIR6482*, *HbMIR6483* and *HbMIR6485* were also strongly expressed (from 2.20 for *HbMIR6482* to 7.44 × 10 for *HbMIR6485* in leaves).

In the same *in vitro* plantlets, statistical analyses revealed a difference in gene expression at tissue level (Table 1). Indeed, three genes *HbMIR166b*, *HbMIR319* and *HbMIR6482* had a relative differential expression level in leaves, bark and roots under untreated conditions. *HbMIR166b* and *HbMIR319* were more strongly expressed in bark while *HbMIR6482* was more strongly expressed in roots. For these three genes, the lowest expression level was found in leaves (Table 1). In addition, seven genes significantly displayed a relative differential expression level, namely *HbMIR159a*, *HbMIR398a*, *HbMIR398b*, *HbMIR408b*, *HbMIR6483*, *HbMIR6484* and *HbMIR6485*. The *MIR398c* transcript was only present in trace form under the majority of conditions.

The regulation of *MIR* genes in response to cold and salinity stresses was shown as the ratio between the mean of three treated *in vitro* plantlets and three untreated *in vitro* plantlets (Table 1). An analysis of the relative expression of the *MIR* genes showed that 8 *MIR* genes were significantly regulated in response to cold (Table 1). In leaves, both *HbMIR159* genes displayed a significant increase in the relative number of transcripts in response to cold while the relative expression of *HbMIR6482* was greatly inhibited. In bark, the relative expression of 3 *MIR* genes (*HbMIR166b*, *HbMIR476* and *HbMIR6482*) was repressed while that of *HbMIR159b* was slightly stimulated. In roots, 5 *MIR* genes (*HbMIR159a*, *HbMIR408a*, *HbMIR408b*, *HbMIR6482* and *HbMIR6484*) displayed a significant reduction in transcripts in response to cold. The drop in temperature led to a drop in transcripts for *HbmiR6482* in the 3 tissues. *HbMIR159a* displayed an expression profile that was antagonistic between leaves and roots. With the exception of *HbMIR159a* and *HbMIR159b*, the significantly regulated *MIR* genes were repressed in response to cold stress.

The *in vitro* plantlets were also subjected to saline stress by watering with a 300 mM NaCl solution rather than water twice a day for 24 h. In response to NaCl, 16 of the 20 genes displayed a significant repression in their level of relative transcript abundance. Only *HbmiRn12* was highly induced (4 to 6 times) in most tissues.

### 2.2. Relative Accumulation of preMIR in Response to Ethylene (ET), Methyl Jasmonate (MeJA) and Wounding in Three-Month-Old Epicormic Shoots

Based on previous studies on budded plant material [31], the effect of ET, MeJA and wounding was studied on three-month-old epicormic shoots. In control plants, the relative level of premiR was highly accumulated for *HbMIR6485* and *HbMIRn13* (1.60 × 10 and 3.92 × 10^2^ respectively) and at low level for *HbMIR156*, *HbMIR319* and *HbMIR398a* (4.42 × 10^−4^, 1.15 × 10^−2^ and 3.61 × 10^−4^, respectively; Table 2). This result was similar to the basal expression profile found in the *in vitro* plantlets. 7 *MIR* genes had a relative expression level that differed significantly between bark and leaves (Table 2). They were *HbMIR159a*, *HbMIR159b*, *HbMIR166b*, *HbMIR319*, *HbMIR396*, *HbMIR398a* and *HbMIR6485*. In comparison, the aforementioned genes were also regulated differently depending on the tissues in the *in vitro* plantlets, apart from *HbMIR396*. Again, the *MIR398c* transcript was only present in trace form under the majority of conditions.

The regulation of *MIR* genes in response to ethylene, MeJA and wounding was shown as the ratio between the mean of three treated *in vitro* plantlets and three untreated *in vitro* plantlets. 3 *MIR* genes were significantly regulated by the ethylene treatment (Table 2). In leaves, only the relative expression of the *HbMIR398a* gene was repressed. In bark, 2 *MIR* genes were negatively regulated by ethylene, and the expression of *HbMIR319* and *HbMIR396* was inhibited (Table 2). Application of methyl jasmonate induced regulation of the relative expression of 6 *MIR* genes in leaves and not in bark (Table 2). The *HbMIR159a*, *HbMIR159b*, *HbMIR166a*, *HbMIR476* and *HbMIR6485* genes displayed a relative increase in transcripts, while *HbMIR408b* displayed a significant large drop in transcripts in response to external methyl jasmonate (Table 2). In response to wounding, 5 genes were significantly regulated (Table 2). In leaves, *HbMIR408a* and *HbMIR408b* displayed a large reduction in their relative expression levels while *HbMIR6482* significantly showed an increase in relative abundance for these transcripts (Table 2). In bark, the *HbMIR166b* and *HbMIR319* genes displayed a significant drop in their relative transcript abundance (Table 2). It should be noted that the *HbMIR319* gene was regulated in the same way in response to both ethylene and wounding.

The response to jasmonate in leaves seemed to differ from the other abiotic stresses. In fact, the expression of 5 *MIR* genes (*MIR159a*, *MIR159b*, *MIR166*, *MIR476* and *MIR6485*) was stimulated during the treatment. MiR159 and miR476 are known to target type MYB transcription factors [17,32] and PPRs (pentatricopeptide repeat proteins) [33], respectively. These targets were not identified during the prediction of targets of miR159 and miR476 in *Hevea*. In contrast, miR166 is known to target proteins of the HD-ZIPIII family in *Arabidopsis* and also in *Hevea* [17,26].

**Figure 1:**
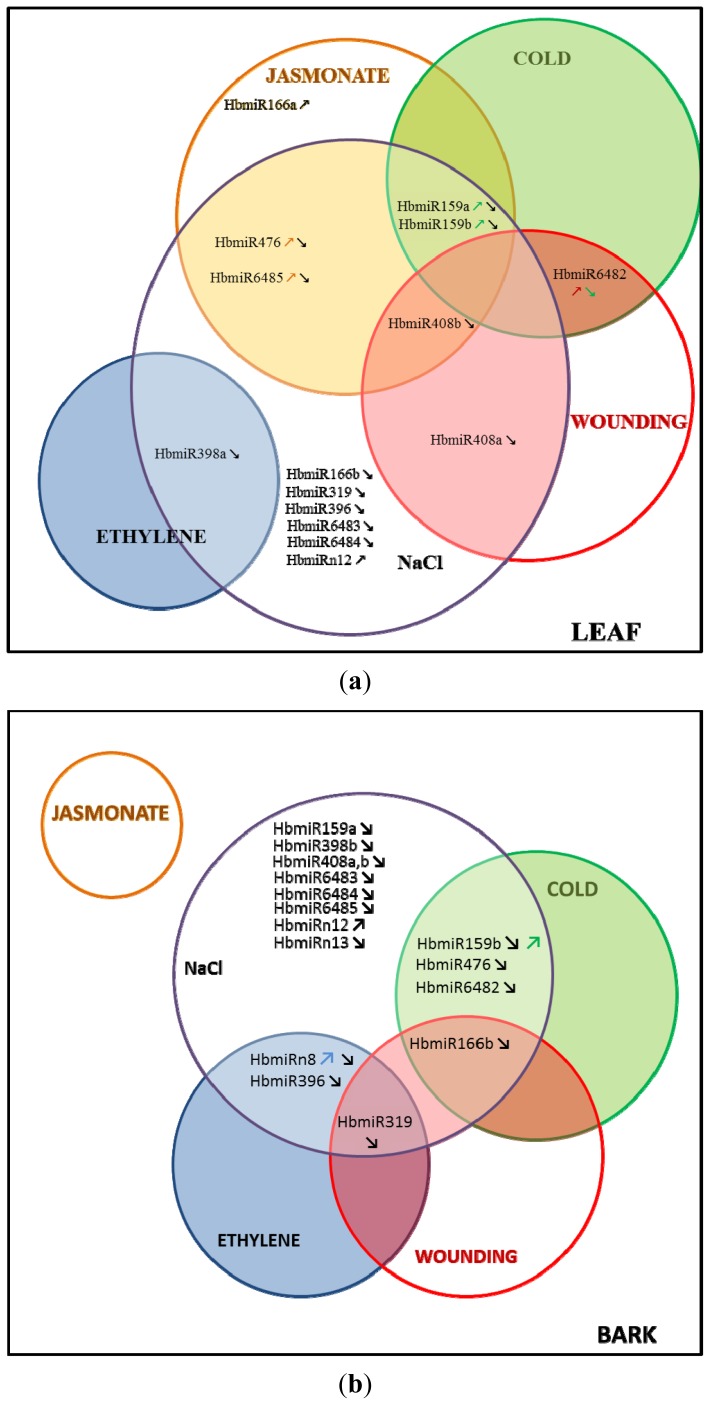
Venn diagram representing the regulation of the *MIR* genes common to all the treatments tested. (**a**): in leaves; and (**b**): in bark.

The expression of *MIR* genes was differentially regulated depending on the stress and the tissue (bark and leaf), suggesting specific response pathways for each type of stress (Figure 1a,b).

In maize, conversely, the comparison of expression pattern of mature miRNAs in plant subjected to saline stress and a viral infection lead the authors to suggest some cross-talk between abiotic and biotic stress [34]. In addition to differential expression depending on the tissue and stress applied, some differences between species are worth noting. In *Hevea brasiliensis*, the response to cold stress differs from that found in *Arabidopsis thaliana* [35]. In *Arabidopsis*, miR166b is accumulated in response to cold stress whereas in *Hevea*, repression of the *HbMIR166b* gene is found. Inhibition of the relative expression of the *HbMIR476* gene in *Hevea* bark shows an expression profile similar to that in poplar [36]. For saline stress, regulation of the expression of microRNAs appears to be specific to each species. In *Hevea*, saline stress led to global repression of the expression of all the *MIR* genes studied, except *HbMIRn12*. In this case, the result suggests that response mechanisms were brought into play by blocking the repression of genes targeted by microRNAs. By contrast, in *Arabidopsis*, accumulation of the mature microRNAs miR156, miR159, miR319 and miR396 and a reduction in the quantity of miR398 are seen [37]. The quantity of miR396 and miR156 is reduced in rice and maize respectively [38]. In poplar, the quantity of miR530a, miR1445, miR1446a-e, miR1447 and miR171l is reduced while that of miR482.2 and miR1450 is increased [36].

The difference observed between *Hevea* and other plants could be explained by post-transcriptional regulations acting between pri-*MIR* transcript and the release of mature miRNA [39]. Moreover, most of studies reported on total mature miRNAs using Northern-blot hybridization highlighting the final products of several *MIR* genes. In our study, the use of real-time RT-PCR allowed identifying the specific expression of each *MIR* gene isoform. The microRNAs newly identified in *Hevea brasiliensis* have a high level of expression compared to that of conserved microRNAs under normal conditions. Different elements tend to show that the new microRNAs (young miRNAs) have little or no function [40]. Although conserved microRNAs are generally more abundant than the new microRNAs [41], an abundance of the latter does not mean greater regulation of the targets. Regulation of the targets of new microRNAs is not affected in mutant transgenic plants in the miRNA biogenesis pathway. The new microRNAs would seem not to be integrated yet in the regulation networks or may only function in a precise spatio-temporal context, or during response to a specific type of biotic or abiotic stress [40].

### 2.3. Analysis of the Co-Regulated Expression of the *HbMIR398a*, *HbMIR398b* and *HbMIR398c* Genes and Their Putative Target Gene Chloroplastic HbCuZnSOD in Response to Abiotic Stress and to Hormone Treatments

**Figure 2:**
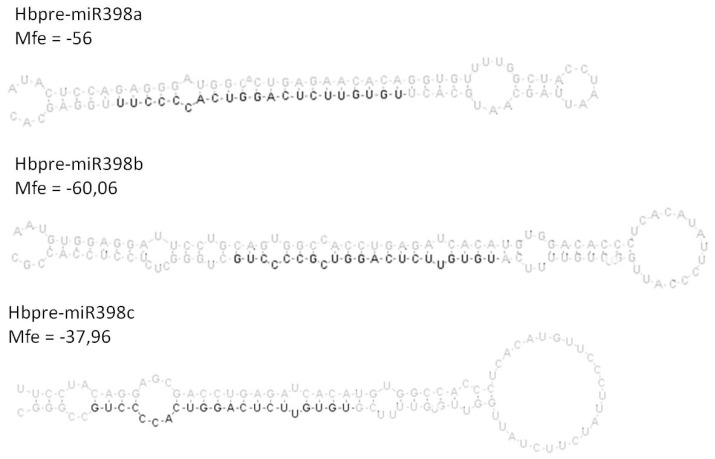
Stem-loop structure of three precursors of *MIR398* in *Hevea brasiliensis*. The mature microRNA sequence is shown in dark.

Three miR/target pairs have been experimentally validated in the rubber tree (miR156/Squamosa promoter binding protein, miR160/ARF and miR398/chloroplastic CuZnSOD) [26]. We chose to monitor expression of the *MIR398/chloroplastic CuZnSOD* pair. HbmiR398 is generated by three *HbMIR398* genes (3 genes, a, b and c; Figure 2).

In order to monitor expression of the *chloroplastic HbCuZnSOD* target, two primer pairs were designed, one flanking the cleavage site and the other at 3′ UnTranslated Region (3′UTR) (Table 3).

In response to abiotic stress, the transcripts of the *chloroplastic HbCuZnSOD* were accumulated significantly in roots in response to cold, in bark and roots in response to saline stress, and particularly after 24 h of treatment revealing that these transcripts are not cleaved by miR398 (Table 4, Tables S1 and S2). When the relative abundance of *chloroplastic HbCuZnSOD* transcripts was visualized (cleaved and non-cleaved with the primers at 3′UTR), the relative expression level for *chloroplastic HbCuZnSOD* transcripts decreased in leaves after 24 h of saline stress. However, that relative expression level rose significantly in response to saline stress in roots after 24 h of saline stress, and in wounded leaves (Table 4).

The relative accumulation of premiRs was analyzed for the three *MIR398* genes. The *MIR398c* transcript was only present in trace form under the majority of conditions. The relative expression of the *MIR398a* and *MIR398b* genes was significantly repressed in response to stress. In fact, in leaves, the relative expression of the *MIR398a* gene was repressed in response to 24 h of saline and ethylene treatment. Likewise, the relative expression of the *MIR398b* gene was repressed in bark and roots after 24 h of saline stress treatment.

**Figure 3:**
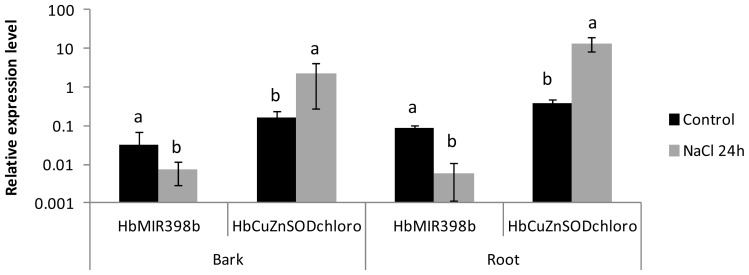
Graph showing the expression values for the *chloroplastic HbCuZnSOD* and *HbMIR398b* genes in response to saline stress in bark and roots after 24 h of treatment. The expression values represent the mean and standard deviation of three treated or untreated plants, for each tissue. Data with the same letter are not significantly different at the level of 5%.

When the relative expression level for the *HbMIR398* gene was compared with the expression data for the target, the significant reduction in the expression of the *HbMIR398b* gene in bark and roots in response to 24 h of saline stress was accompanied by a significant increase in the relative expression level for the non-cleaved *chloroplastic HbCuZnSOD* transcripts (Figure 3).

Co-expression of *MIR398* and of the gene encoding chloroplastic CuZnSOD was analyzed in response to several treatments. This enzyme takes part in ROS detoxification through the transformation of two superoxide ions H_2_O_2_ and H_2_O. In *Arabidopsis*, miR398 targets cytosolic and chloroplastic superoxide dismutase (CSD1 and CSD2, [22]). Under oxidative stress conditions (heavy metal, strong sunlight and methyl viologen treatments), negative regulation of CSD1 and CSD2 by miR398 is halted in leaves, leading to an accumulation of enzymes and a drop in toxic free radicals for the cell. Tolerance of oxidative stress is consequently increased [22]. Interestingly, the *chloroplastic CuZnSOD* is the only one to have been validated in *Hevea* for miR398, the cytosolic form was not predicted to be targeted by miR398 and the absence of cleavage was experimentally validated [26]. Some other targets have been predicted for this microRNA but have yet to be experimentally validated [26]. Co-regulation was only visible in response to saline stress. A reduction in the expression of the *HbMIR398b* gene was associated with an accumulation of *chloroplastic HbCuZnSOD* transcripts in bark and roots but not in leaves. It is noteworthy to specify that the bark of the young *Hevea in vitro* plantlets was also chlorophyllous. For root tissues, other plastid organelles might be present. In addition, the level of expression for the *MIR398* genes was not always negatively correlated with the level of *chloroplastic HbCuZnSOD* transcripts, which has already been found in other species and suggests the existence of two regulation mechanisms, dependent on and independent of miR398 [42].

## 3. Experimental Section

### 3.1. Plant Material and Treatments

*In vitro* plantlets were regenerated by indirect secondary somatic embryogenesis from maternal inner integument of immature fruits from rubber clone PB 260 [43]. These *in vitro* plantlets were acclimatized and reared in the greenhouse for one year at a temperature of 28 °C. At that stage, the treatments were applied on three biological replicates. For cold stress, the plants were placed at 4 °C for 12 h. Saline stress was applied to the *in vitro* plantlets by watering with a 300 mM solution of NaCl rather than just water twice a day for the duration of the treatment (24 h).

Three-month-old epicormic shoot from grafted plants were used for ethylene (ET), methyl-jasmonate (MeJA) and wounding treatments. Plants from the *Hevea* clone PB 260 were grafted onto rootstock GT1. This plant material included a mature growth unit corresponding to a three-month-old epicormic shoot after cutting back. The treatment was applied for 4 h on the whole plant at 8:00 a.m. For application of the ethylene and methyl jasmonate treatments, each plant was placed in a hermetically sealed plexiglas box with a volume of 300 L leaving the doors open for 24 h prior to treatment to limit the effects of transport or any wounds. For application of the ethylene treatment, the gas was injected to obtain an ethylene concentration of 1 ppm (300 μL of pure ethylene) in the box. For the methyl jasmonate treatment, 20 μL of methyl jasmonate solution (>95%) (Sigma, St. Louis, MO, USA) was diluted in 500 μL of absolute ethanol and placed on Whatman paper inside the box, in order to release the methyl jasmonate in gas form. For these two stress conditions, samples were taken 4 h after treatment. The plants used as controls for these treatments were also placed in a hermetically sealed box without gas injection. In that way, it was possible to eliminate the impact of the box when comparing the two treatments (control and treated). For the wounding treatment, the plants were wounded on their leaves by applying pressure every 2 cm with serrated tweezers and on the bark by making cuts with a scalpel 1 cm apart on the stem of the scion.

Table 1 summarizes the different treatments applied to the rubber *in vitro* and grafted plants. Some leaf, bark and root samples were taken after 12 h for the *in vitro* plantlets, and only leaves and bark for the budded plants whose root system was not clonal. All the samples were immersed directly in liquid nitrogen after being taken and were stored at −80 °C pending RNA extraction.

### 3.2. Extraction and Purification of Total RNAs

The samples (leaf, bark and root) were ground in liquid nitrogen. The resulting fine powder was placed on ice in an extraction buffer (25 mM guanidium isothiocyanate 5M NaAc, 0.88% of sarcosine, 0.9% of polyvinylpyrrolidone (PVP) and 1% of β-mercapto-ethanol). The samples were mixed for 30 s to optimize membrane lysis. An initial centrifugation (30 min at 4 °C at 10,000 *g*) was applied to precipitate cell debris. The supernatant was deposited on a caesium cushion (24 mM NaAc, 45.6 mM CsCl). The tubes were then balanced and centrifuged for 20 h at 20 °C at 25,000 rpm in a Beckmann Coulter ultracentrifuge (L7, Beckmann Coulter, Brea, CA, USA) using the SW41 rotor (Beckmann Coulter). Only dense RNAs were able to cross the caesium chloride cushion. After 20 h, the supernatant was removed by pipetting, leaving 1 cm height of solution, which was eliminated by tipping up the tube. This precaution was taken to avoid contamination by genomic DNA. The pellet was dried for 5 min then rinsed in 70% ethanol then taken up in 200 μL of sterile water. The RNAs obtained were quantified then checked by electrophoresis (Mini-Sub Cell, Biorad, Richmond, CA, USA) on agarose gel (1% *w*/*v*, 1× Tris Acetate Ethylenediaminetetraacetic acid) and stained with ethidium bromide for visualization under ultraviolet. The RNAs were then aliquoted and stored at −80 °C.

### 3.3. Checking for the Presence of Genomic DNA and DNAse Treatment

A polymerase chain reaction (PCR) was carried out to check for the presence of genomic DNA in the samples of extracted total RNA. Amplification was carried out in a final volume of 50 μL. This was done with a volume of RNA corresponding to 50–100 ng to which was added a reaction mixture comprising 10× reaction buffer (10 mM Tris, pH = 8.3), MgCl_2_ (1.5 mM), dNTP (0.04 mM), gene primers encoding actin (Forward: (5′-TCCATAATGAAGTGTGATGT-3′, Reverse: (5′-GGACCTGACTCGTCATACTC-3′, chosen astride an intron) (0.04 μM) and Taq polymerase (1 U/μL). A sample of *Hevea* genomic DNA was used as the positive control. The samples were incubated for 1 min and 30 s at 94 °C followed by 40 cycles, each composed of 30 s of denaturing at 94 °C, 30 s of hybridization at 58 °C and 90 s of elongation at 72 °C, with the reaction ending on a final elongation of 10 min at 72 °C. The amplification products were deposited on an agarose gel (1% *w*/*v*, 1× Tris Acetate Ethylenediaminetetraacetic acid) then stained with ethidium bromide. The RNA samples that proved to be contaminated by genomic DNA were treated by digestion with DNase (Turbo DNA-FREE kit from Ambion, Life technologies, Carlsbad, CA, USA). The reaction took place from 8 ng of total RNAs in a final volume of 40 μL composed of DNase buffer (10×, 4 μL, Ambion), DTT (0.1 M, 2 μL, Invitrogen, Life technologies, Carlsbad, CA, USA), RNase inhibitor (RiboLock, Fermentas, Burlington, ON, Canada, 40 U/μL) and TURBO DNase (2 U/μL). The samples were then incubated for 15 min at 37 °C. Next, a DNase inactivator (TURBO DNase Inactivation Reagent, Ambion) was added to the RNA samples. Enzyme inactivation took place for 2 min at ambient temperature by mixing the tubes. The supernatant containing the purified RNAs was recovered by centrifugation (12,000 *g*, 2 min, 4 °C).

### 3.4. cDNA Synthesis and Real-Time PCR

A microgram of total RNAs was used to synthesize some cDNAs in a reaction volume of 20 μL using RevertAid™ M-MuLV reverse transcriptase (MBI, Fermentas, Burlington, Canada) following the supplier’s recommendations. The primers used to amplify the stem-loop structures are listed in Table 2. The PCR conditions comprised a denaturing cycle at 95 °C for 2 min, then 45 cycles comprising denaturing at 95 °C for 20 s and a hybridization and elongation stage at 60 °C for 20 s. The PCR reaction took place in a volume of 6 μL containing 2 μL cDNA diluted 25 times, 1 μL of primers at 5 μM, and 3 μL of 2× SYBR green mixture (LightCycler^®^ 480 SYBR Green I Master, Roche Applied Sciences, Bâle, Switzerland). All the primers were validated by generating a standard curve from the dilution in series of 8 points, in triplicate, to calculate the efficiency of each primer pair (Table 3). In addition, amplification specificity was checked by generating the melting curves that needed to display a unique peak, and all PCR products were checked by sequencing. Relative quantification was carried out with Light Cycler 480 software (version 1.5.0, Roche Applied Sciences, Bâle, Switzerland), using *RH2b* [44] as the reference.

### 3.5. Statistical Analysis

The statistical analyses (analysis of variance followed by a Fisher comparison of means test) were done with XLSTAT (version 2011.4.02, Addinsoft, Paris, France) using expression data normalized by the LOG10(X) function. It was considered as up-regulation when the ratio was >1.0 and down-regulation when the ratio was <1.0. The p value corresponds to the Fisher test of the ANOVA.

## 4. Conclusions

Studying the regulation of *MIR* genes under harvesting stress (tapping, ethephon stimulation) and ROS-induced TPD in mature rubber trees remains difficult. However, some recent studies revealed that only one *MIR* genes is differentially regulated upon TPD. The *MIR159b* gene was shown up-regulated upon TPD occurrence [24]. The expression of this gene was increased in response to cold in leaves and bark, and in response to the jasmonic acid treatment in leaves of juvenile plantlets. Conversely, it was inhibited in all the tissues in response to salinity.

In order to get a full understanding of mechanisms involved in latex production and TPD syndrome, a complete validation of miRNA/target messenger pairs is first needed using by high throughput “degradome” analysis [45]. Combination of analyses on juvenile and mature plant materials will help developing model of *MIR* gene regulations under abiotic stress and further characterization of the TPD-regulated miRNAs and their targets.

## Supplementary Information



## Figures and Tables

**Table 1:** Relative premiR abundance of 13 conserved *MIR* genes and 7 *MIR* genes newly identified in *Hevea brasiliensis* in the leaves, bark and roots of one-year-old *in vitro* plantlets in response to cold and saline (NaCl) treatments compared with non-treated plants (C: control), nc: not calculated. Data with the same letter are not significantly different at the level of 5%.

Gene	Tissue	Expression value for control	Cold/C	*p*-value	NaCl/C	*p*-value
*HbpremiR156*	leaf	1.72 × 10^−3^^a^	1.79	0.52	0.17	0.08
	bark	1.80 × 10^−3^^a^	0.91	0.72	0.20	0.08
	root	2.63 × 10^−3^^a^	0.70	0.39	0.39	0.13

*HbpremiR159a*	leaf	4.68 × 10^−2^^b^	3.45	0.008	0.04	0.002
	bark	7.45 × 10^−2^^a^	1.25	0.22	0.17	<0.001
	root	5.94 × 10^−2^^a^,^b^	0.32	0.02	0.10	0.01

*HbpremiR159b*	leaf	1.56 × 10^−1^^a^	4.06	0.004	0.05	0.003
	bark	2.21 × 10^−1^^a^	1.59	0.03	0.23	<0.0001
	root	1.94 × 10^−1^^a^	0.44	0.23	0.11	0.02

*HbpremiR166a*	leaf	1.19 × 10^−2^^a^	1.38	0.38	2.08	0.08
	bark	1.78 × 10^−2^^a^	2.00	0.18	0.70	0.16
	root	4.16 × 10^−2^^a^	0.60	0.62	2.76	0.13

*HbpremiR166b*	leaf	2.21 × 10^−1^^c^	1.34	0.31	0.20	0.01
	bark	5.20 × 10^0^^a^	0.64	0.01	0.14	0.003
	root	9.67 × 10^−1^^b^	0.48	0.39	1.01	0.80

*HbpremiR319*	leaf	5.73 × 10^−3^^c^	0.29	0.08	0.23	0.02
	bark	9.22 × 10^−2^^a^	1.30	0.90	0.07	<0.0001
	root	1.76 × 10^−2^^b^	nc	–	nc	–

*HbpremiR396*	leaf	4.73 × 10^−1^^a^	1.34	0.28	0.10	0.002
	bark	1.41 × 10^0^^a^	0.93	0.61	0.17	<0.0001
	root	2.33 × 10^0^^a^	0.28	0.19	0.12	0.05

*HbpremiR398a*	leaf	6.93 × 10^−3^^a^	0.34	0.12	0.21	0.02
	bark	2.27 × 10^−3^^a^,^b^	0.23	0.31	0.49	0.64
	root	6.78 × 10^−4^^b^	0.47	0.39	0.10	0.20

*HbpremiR398b*	leaf	3.27 × 10^−2^^b^	0.95	0.92	0.87	0.61
	bark	8.68 × 10^−2^^a^	0.52	0.15	0.07	0.01
	root	3.19 × 10^−2^^b^	0.62	0.34	0.15	0.03

*HbpremiR398c*	leaf	trace	nc	–	nc	–
	bark	trace	nc	–	nc	–
	root	trace	nc	–	nc	–

*HbpremiR408a*	leaf	8.47 × 10^−1^^a^	0.47	0.20	0.10	<0.0001
	bark	1.50 × 10^0^^a^	0.23	0.11	0.07	<0.001
	root	1.48 × 10^0^^a^	0.22	0.02	0.06	0.01

*HbpremiR408b*	leaf	1.60 × 10^0^^b^	0.48	0.21	0.12	<0.0001
	bark	3.54 × 10^0^^a^	0.22	0.11	0.08	0.01
	root	2.75 × 10^0^^a^,^b^	0.22	0.01	0.08	0.001

*HbpremiR476*	leaf	2.71 × 10^−2^^a^	0.66	0.39	0.16	0.01
	bark	2.84 × 10^−2^^a^	0.36	0.01	0.37	0.002
	root	8.49 × 10^−2^^a^	0.20	0.11	0.41	0.23

*HbpremiR6482*	leaf	2.20 × 10^0^^c^	0.09	0.001	0.30	0.06
	bark	9.86 × 10^0^^b^	0.21	0.002	0.45	0.01
	root	7.06 × 10^a^	0.05	0.004	0.05	0.003

*HbpremiR6483*	leaf	5.47 × 10^a^	0.59	0.50	0.03	0.002
	bark	1.56 × 10^a^	0.70	0.43	0.18	0.01
	root	5.54 × 10^−1^^b^	1.05	0.72	0.51	0.59

*HbpremiR6484*	leaf	7.17 × 10^−1^^a^	0.70	0.26	0.22	0.003
	bark	7.47 × 10^−1^^a^	0.65	0.06	0.55	0.02
	root	3.47 × 10^−1^^b^	0.25	0.02	0.35	0.05

*HbpremiR6485*	leaf	7.44 × 10^a^	0.73	0.49	0.03	0.000
	bark	3.14 × 10^a^	0.82	0.77	0.22	0.02
	root	6.06 × 10^0^^b^	0.73	0.14	0.27	0.01

*HbpremiRn11*	leaf	trace	nc	–	nc	–
	bark	trace	nc	–	nc	–
	root	trace	nc	–	nc	–

*HbpremiRn12*	leaf	7.26 × 10^−2^^a^	1.25	0.50	6.45	<0.0001
	bark	8.80 × 10^−2^^a^	0.75	0.29	5.93	<0.001
	root	1.47 × 10^−1^^a^	0.48	0.10	4.17	0.01

*HbpremiRn13*	leaf	3.77 × 10^2^^a^	1.17	0.60	0.34	0.11
	bark	3.41 × 10^2^^a^	1.17	0.74	0.46	0.001
	root	2.65 × 10^2^^a^	0.81	0.51	1.90	0.06

**Table 2:** Relative premiR abundance of 13 conserved *MIR* genes and 7 *MIR* genes newly identified in *Hevea brasiliensis* in the leaves and bark collected on 3-month-old epicormic shoot of budded plants in response to ethylene (ET), methyl jasmonate (MeJA) and wounding (W) compared with non-treated plants (C: control), nc: not calculated. Data with the same letter are not significantly different at the level of 5%.

Gene	Tissue	Expression valuefor control	ET/C	*p*-value	MeJA/C	*p*-value	W/C	*p*-value
*HbpremiR156*	leaves	4.42 × 10^−4^^a^	1.02	0.94	6.23	0.06	2.92	0.68
	bark	6.80 × 10^−4^^a^	1.63	0.26	0.66	0.62	1.68	0.35

*HbpremiR159a*	leaves	2.34 × 10^−1^^a^	1.22	0.91	1.40	0.04	1.04	0.88
	bark	4.41 × 10^−2^^b^	0.40	0.30	0.75	0.99	0.33	0.20

*HbpremiR159b*	leaves	6.28 × 10^−1^^a^	0.81	0.38	1.64	0.02	0.76	0.14
	bark	1.96 × 10^−1^^b^	0.50	0.35	0.86	0.92	0.41	0.22

*HbpremiR166a*	leaves	4.02 × 10^−2^^a^	0.49	0.39	2.84	0.05	0.98	0.84
	bark	1.62 × 10^−2^^a^	0.35	0.17	1.00	0.99	1.06	0.72

*HbpremiR166b*	leaves	5.98 × 10^0^^a^	1.50	0.24	2.45	0.22	1.78	0.07
	bark	1.00 × 10^−1^^b^	1.08	0.67	1.81	0.15	0.32	0.03

*HbpremiR319*	leaves	2.80 × 10^−1^^a^	0.73	0.63	1.46	0.33	0.87	0.83
	bark	1.15 × 10^−2^^b^	0.31	0.03	1.28	0.41	0.18	0.02

*HbpremiR396*	leaves	2.87 × 10^0^^a^	0.62	0.20	3.19	0.09	0.57	0.22
	bark	4.80 × 10^−1^^b^	0.27	0.02	0.50	0.13	0.43	0.07

*HbpremiR398a*	leaves	3.61 × 10^−4^^b^	0.22	0.05	0.69	0.53	1.85	0.34
	bark	1.66 × 10^−2^^a^	3.59	0.08	3.38	0.21	2.99	0.19

*HbpremiR398b*	leaves	1.89 × 10^−3^^b^	0.52	0.67	0.09	0.41	0.05	0.27
	bark	5.01 × 10^−2^^a^,^b^	15.19	0.09	0.61	0.46	4.66	0.60

*HbpremiR398c*	leaves	trace	0.91	0.82	nc	–	0.04	0.16
	bark	trace	nc	–	nc	–	nc	–

*HbpremiR408a*	leaves	3.49 × 10^0^^a^	0.40	0.61	0.05	0.21	0.04	0.002
	bark	1.29 × 10^0^^a^	0.50	0.64	0.002	0.52	0.05	0.62

*HbpremiR408b*	leaves	5.40 × 10^0^^a^	0.42	0.55	0.02	0.01	0.04	0.001
	bark	2.52 × 10^0^^a^	0.73	0.30	0.003	0.30	0.05	0.91

*HbpremiR476*	leaves	1.49 × 10^−1^^a^	0.84	0.52	17.93	0.001	0.98	0.73
	bark	1.73 × 10^−2^^a^	0.26	0.10	6.18	0.06	0.32	0.30

*HbpremiR6482*	leaves	4.41 × 10^0^^a^	1.14	0.60	1.60	0.26	7.19	0.05
	bark	1.63 × 10^0^^a^	0.62	0.48	1.32	0.53	2.68	0.14

*HbpremiR6483*	leaves	5.56 × 10^0^^a^	2.97	0.18	4.14	0.13	0.81	0.85
	bark	8.63 × 10^0^^a^	1.13	0.67	1.34	0.69	0.68	0.53

*HbpremiR6484*	leaves	6.10 × 10^−1^^a^	0.92	0.65	1.67	0.14	0.48	0.12
	bark	8.92 × 10^−1^^a^	0.99	1.00	1.15	0.72	0.73	0.41

*HbpremiR6485*	leaves	1.60 × 10^b^	1.19	0.58	2.62	0.004	0.61	0.11
	bark	3.00 × 10^a^	1.00	0.94	0.82	0.44	0.52	0.06

*HbpremiRn11*	leaves	trace	nc	–	3.52	0.08	1.35	0.98
	bark	trace	0.97	0.93	0.56	0.24	0.60	0.39

*HbpremiRn12*	leaves	6.19 × 10^−2^^a^	1.23	0.65	1.51	0.12	7.12	0.13
	bark	6.13 × 10^−2^^a^	1.20	0.58	0.84	0.96	1.42	0.44

*HbpremiRn13*	leaves	3.16 × 10^2^^a^	1.23	0.39	1.24	0.55	1.41	0.96
	bark	3.92 × 10^2^^a^	1.85	0.16	0.91	0.55	1.85	0.63

**Table 3:** List of primers and their sequences used for *MIR* gene expression analysis by RT-PCR. All primers are presented from 5′ to 3′ end.

Pre-microRNA	Forward primer	Reverse primer	PCR efficiency
*Hbpre-miR156*	TGGTGATGTTGTTGACAGAAGATAGAGAGC	GCACAAAGGAGTGAGATGCAGAGTCC	1.79
*Hbpre-miR159a*	GGTTAAGAAGTGGAGCTCCTTGAAGTC	GCTCCCTTCAATCCAAACAAGGATC	1.958
*Hbpre-miR159b*	GTGGAGCTCCTTGAAGTCCAATAGAGG	AGAGCTCCCTTCAATCCAAACAAGG	1.881
*Hbpre-miR166a*	TTCTTTTTGAGGGGAATGTTGTCTGG	GGAATGAAGCCTGGTCCGAGGAG	1.820
*Hbpre-miR166b*	GGGGAATGTTGTCTGGTTCGATG	TCAAATCAAACCCTGTTGGGGG	1.738
*Hbpre-miR319*	CCAGTCACGGTGGGCAATGGG	GGAGCTCCCTTCAGTCCAAGTACAGG	1.847
*Hbpre-miR396*	TGACCCTCTTCGTATTCTTCCACAGC	CCCACAGCTTTATTGAACCGCAAC	1.782
*Hbpre-mir398a*	TGAGAACACAGGTGTTTTGGCTACC	GTGCTCCAAAGGGGTGACCTGAG	1.879
*Hbpre-mir398b*	ACCTGAGATCACATGTGGACACCC	GCGGTGGAGGAGAGCCCAG	1.939
*Hbpre-mir398c*	TGGCCACCCTCACATGTTCCC	CCGGCAGGGGTGACCTGAG	1.965
*Hbpre-miR408a*	ACTGGGAACAGGCAGAGCATGG	GCCACAAGCCAGGGAAGAGGC	1.723
*Hbpre-miR408b*	GACATACAAAGACTGGGAACAGGCAG	GCCACAAGCCAGGGAAGAGGC	1.792
*Hbpre-miR476*	GCCTTGTATGTTTCATTTAGTAATCCTTCT	GATAATCCTTCTATGCAAAGTCTTTTATGC	1.732
*Hbpre-miR6482*	ACCAGGAACTGGTATCAACCCAGC	TGCTACCAATGAATCGGACCCACC	1.837
*Hbpre-miR6483*	CAGTAAATAGCAGTATCGTGGATAGGG	GTCCAATCATTGATCCTGAAAATTTCTAC	1.828
*Hbpre-miR6484*	TGGATTGGAGCCCAATACTGTGAC	CTGCTCCATTGATTTTACCATCTATGC	1.873
*Hbpre-miR6485*	ACCTAGGATGTAGAAGAGCATAAC	ACTACATGAGTGGATATATAGGAATCC	1.787
*Hbpre-miRn11*	GTATCAACGCAGATGTGCCGCC	CCCCAGCCAAACTCCCCACC	1.828
*Hbpre-miRn12*	AGCTTTCACCCAATAACCTTTGCAGT	GCTCTTCCAATTCCTATCCAAAGTGGT	1.78
*Hbpre-miRn13*	TGTGTTGGCCTTCGGGATCGG	CGAATGCCCCCGACTGTCCC	1.889
*Hb-RH2b*	GAGGTGGATTGGCTAACTGAGAAG	GTTGAACATCAAGTCCCCGAGC	1.68
*HbCuZnSOD (flanking miRNA site)*	GCTCTATCTCTCGCCGCCGCCTCC	CCGCAATTGTTGCTTCTGCC	1.785
*HbCuZnSOD (3*′*UTR)*	TGGCAGAAGCAACAATTGCGG	GCAGGGAACAATGGCTGCC	2

**Table 4:** Expression profiles of the *HbMIR398a*, *HbMIR398b* and *HbMIR398c* genes and their *chloroplastic HbCuZnSOD* target by real time-PCR in leaves, bark and roots of one-year-old *Hevea brasiliensis in vitro* plantlets in response to a positive cold, NaCl, ethylene, methyl jasmonate (MeJA) and wounding treatments. The statistically significant ratios (*p*-value < 0.05; Tables S1 and S2) with a value over 1 (**in red**) and under 1 (**in green**), represent over-expression and under-expression, respectively, in response to the treatments. The non-significantly regulated genes are shown **in yellow** (nc: not calculated).

Treatment	Tissue	Ratio of relative transcript accumulation (Treated/Control)				

		chloro CuZnSOD (flanking miRNA site)	chloro CuZnSOD (3′UTR)	*preMIR398a*	*preMIR398b*	*preMIR398c*
Cold	leaf	2.71	3.76	0.34	0.95	nc
	bark	8.98	8.90	0.23	0.52	nc
	root	3.41	1.46	0.47	0.62	nc

NaCl	leaf	0.84	0.29	0.21	0.87	nc
	bark	13.99	5.21	0.49	0.07	nc
	root	37.45	17.83	0.1	0.15	nc

Ethylene	leaf	nc	0.42	0.22	0.52	0.91
	bark	0.49	0.32	3.59	15.19	nc

MeJA	leaf	1.47	nc	0.69	0.09	nc
	bark	0.79	1.00	3.38	0.61	nc

Wounding	leaf	2.61	7.07	1.85	0.05	0.04
	bark	0.85	0.88	2.99	4.66	nc
