# Supplementary material for: Regulation of MIR Genes in Response to Abiotic Stress in Hevea brasiliensis
Source: Int J Mol Sci. 2013 Sep 27;14(10):19587–604. doi: 10.3390/ijms141019587 (PMC3821574; doi:10.3390/ijms141019587)
Supplement: Supplementary file 1 [file ijms-14-19587-s001.pdf]

Supplementary Information

Table S1. Expression profiles of the *chloroplastic CuZnSOD* gene by RT-PCR in response to cold and saline stress. “3'UTR”: 3' Untranslated Region.

Treatment	Tissue	Means and *p*-value	*chloroCuZnSOD* (flanking miRNA site)	*chloroCuZnSOD* (3'UTR)
Cold	leaf	mean control	0.76	0.11
		mean cold	2.07	0.41
		p-value	0.83	0.99
	bark	mean control	0.16	0.04
		mean cold	1.46	0.32
		p-value	0.10	0.14
	root	mean control	0.37	0.05
		mean cold	1.25	0.07
		p-value	0.04	0.62
NaCl	leaf	mean control	0.76	0.11
		mean NaCl 24 h	0.64	0.03
		p-value	0.46	0.01
	bark	mean control	0.16	0.04
		mean NaCl 24 h	2.27	0.19
		p-value	0.03	0.21
	root	mean control	0.37	0.05
		mean NaCl 24 h	13.78	0.89
		p-value	0.00	0.00

Table S2. Expression profiles of the *chloroplastic CuZnSOD* gene by RT-PCR in response to ethylene, jasmonate and wounding treatments. "nd": not determined; "3'UTR": 3' UnTranslated Region.

Treatment	Tissue	Means and *p*-value	*chloroCuZnSOD* (flanking miRNA site)	*chloroCuZnSOD* (3'UTR)
Ethylene	leaf	mean control	1.03	0.16
		mean ethylene	nd	0.07
		p-value	nd	0.16
	bark	mean control	1.55	0.22
		mean ethylene	0.76	0.07
		p-value	0.73	0.34
Jasmonate	leaf	mean control	1.03	0.16
		mean MeJA	1.51	nd
		p-value	0.24	nd
	bark	mean control	1.55	0.22
		mean MeJA	1.23	0.21
		p-value	0.93	0.72
Wounding	leaf	mean control	2.26	0.59
		mean wounding	5.91	4.16
		p-value	0.08	0.02
	bark	mean control	1.55	0.22
		mean wounding	1.32	0.19
		p-value	0.92	0.73

© 2013 by the authors; licensee MDPI, Basel, Switzerland. This article is an open access article distributed under the terms and conditions of the Creative Commons Attribution license (http://creativecommons.org/licenses/by/3.0/).
